# Fructose metabolism and its role in pig production: A mini-review

**DOI:** 10.3389/fnut.2022.922051

**Published:** 2022-07-29

**Authors:** Jiahao Xie, Shiyi Shi, Yucheng Liu, Shaoshuai Wang, Shahid Ali Rajput, Tongxing Song

**Affiliations:** ^1^Department of Animal Nutrition and Feed Science, College of Animal Science and Technology, Huazhong Agricultural University, Wuhan, China; ^2^Faculty of Veterinary and Animal Sciences, Muhammad Nawaz Shareef University of Agriculture Multan, Multan, Pakistan

**Keywords:** fructose, pig production, biomedical model, intestine, liver

## Abstract

Epidemiological studies have shown that excessive intake of fructose is largely responsible for the increasing incidence of non-alcoholic fatty liver, obesity, and diabetes. However, depending on the amount of fructose consumption from diet, the metabolic role of fructose is controversial. Recently, there have been increasing studies reporting that diets low in fructose expand the surface area of the gut and increase nutrient absorption in mouse model, which is widely used in fructose-related studies. However, excessive fructose consumption spills over from the small intestine into the liver for steatosis and increases the risk of colon cancer. Therefore, suitable animal models may be needed to study fructose-induced metabolic changes. Along with its use in global meat production, pig is well-known as a biomedical model with an advantage over murine and other animal models as it has similar nutrition and metabolism to human in anatomical and physiological aspects. Here, we review the characteristics and metabolism of fructose and summarize observations of fructose in pig reproduction, growth, and development as well as acting as a human biomedical model. This review highlights fructose metabolism from the intestine to the blood cycle and presents the critical role of fructose in pig, which could provide new strategies for curbing human metabolic diseases and promoting pig production.

## Introduction

Overwhelming evidence has established a strong causal link between excessive fructose intake and metabolic diseases (1). For instance, non-alcoholic-fatty liver disease (NAFLD) is currently the most prevalent liver disease worldwide (2), and excessive consumption of fructose intake is considered to boost diseases (3–5). However, the role of fructose is multifactorial. Diets low in fructose increase the length of the intestines and the height of intestinal villi (6), contributing to weight gain and promoting growth, which suggests the potential and beneficial role of fructose with appropriate concentration. But excessive fructose consumption spills over from the small intestine into the liver, causing steatosis and increasing the risk of colon cancer (7–12). Therefore, it is necessary to clarify the critical role of fructose metabolism and its relationship with metabolic diseases.

Animal models are widely employed for studying fructose-induced metabolic changes. Mice fed a high fructose diet (HFD), such as high fructose corn syrup (HFCS), and conditional genetic mouse models help to address fructose-induced metabolic disorders (13, 14). Notably, pig is well-known as a biomedical model because pig is more similar to human. As omnivores, humans and pigs have a large number of similarities in anatomy, physiology, metabolism, and pathology, e.g., they have similar gastrointestinal anatomy and function, pancreas morphology, and metabolic regulation (15). In addition, pork accounts for more than one-third of meat produced worldwide (16), but there are still some problems such as low growth efficiency in the pig industry.

Currently, some important reviews have summarized the progress in the research on fructose metabolism (11, 17–24). However, fructose consumption and its metabolic role in pig are still poorly understood. Hence, this review will focus on the characteristics and metabolism of fructose and its applications in pig reproduction, growth, and development. The review will also highlight the pig biomedical models in fructose metabolism.

## Characteristics and beneficial function of fructose in physiology

### Characteristics of fructose: A low glycemic sugar

Fructose has been traditionally viewed as a simple 6-carbon monosaccharide found widely in fruit, honey, and some vegetables (17). Over the past few decades, the consumption of soft drinks, especially carbonated drinks, has increased significantly, which are sweetened with sweeteners containing a high percentage of fructose (25, 26).

Fructose is added to beverages and foods in the form of sucrose (50% fructose) or the industrial product high fructose corn syrup (usually 55% fructose) (17). Both fructose and glucose are well absorbed from the intestine into the blood cycle. Admittedly, glucose, not fructose, is the predominant circulating sugar in animals (17), which reflects the innate differences in mammalian glucose and fructose metabolism in circulating blood levels. The normal fasting blood glucose concentration in peripheral blood is 5 mM, while under fasting conditions, the circulating concentration of fructose is about 0.02 mM (27, 28). Early studies reported the average plasma fructose concentration of fructose-fed mice was 1.5 mM, while that of starch-fed mice was 0.23 mM (29). Furthermore, glucose is the main fuel for most tissues and cell types. In contrast, the fructose ingested from sucrose is quickly removed by intestines and liver, converted into glucose and its polymer storage form, glycogen, or fatty acids, and stored in the form of triglycerides in the liver (17). It is of note that fructose has a low glycemic index. Several pieces of evidence have established that the hyperglycemic effect of fructose is much weaker than glucose (30). Thus, the difference between fructose and glucose may suggest the unique role, metabolism, and biological function of fructose.

### Fructose functions as a growth-promotor in mouse model

Due to the decreased concentration gradient of fructose in intestine, namely “first-pass” effect, the fructose is potentially related to regulating intestinal growth and development (31). Strikingly, gavaging of 15% fructose for < 3 months will increase the energy intake, weight gain, and growth of mice (32, 33). Similar results are found by feeding mice with 30% fructose solution for 8 weeks (34). However, when mice were fed with 60% fructose for 6 weeks, the incidence of insulin resistance increased (35). Another study reports that 60% of fructose gavage increases cholesterol, triglycerides, blood glucose levels, and liver lipid peroxidation (36). Interestingly, an administration of low concentration of fructose (~15%) gavaging for 30 weeks results in obesity and liver cirrhosis (37). These observations provide evidence that appropriate dose and time of fructose treatment may enhance the length of the small intestine and its absorption of nutrition, indicating the complexity of metabolic flux.

## Cellular metabolism of fructose with normal and excessive consumption in intestine and liver

### Digestion and absorption of fructose from lumen to intestine

**Figure 1:**
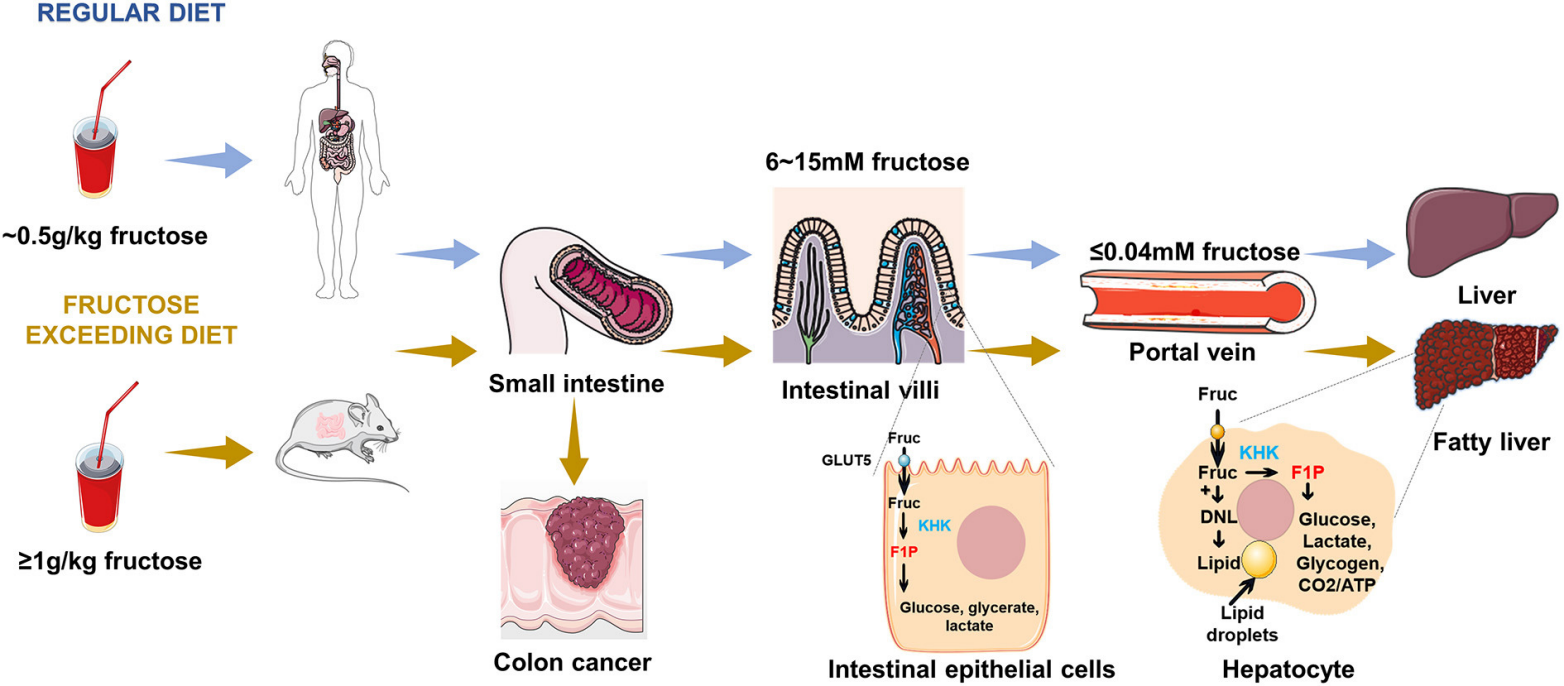
Fructose metabolism in the gut and liver. Following ingestion of fructose, fructose is absorbed into intestinal epithelial enterocytes. Under fasting conditions, the fructose concentration in the small intestine is in the range of 6 ~ 15 mM, while fructose concentration in the portal vein is < 0.04 mM. A portion of this fructose is phosphorylated by KHK within enterocyte and is converted to glucose, lactate, glycerate, and other organic acids, which transport from the portal vein to the liver. Fructose reaching the liver is efficiently extracted by hepatocytes and phosphorylated by KHK, where it can be used for glucose production, lipogenesis, glycogen synthesis, and energetic purposes. An excessive fructose diet can lead to colon cancer, fatty liver, and other related metabolic diseases.

Following oral ingestion, fructose travels through the gastrointestinal tract into the small intestine, where it is passively absorbed from the intestinal lumen mainly by the hexose transporter SLC2A5, also known as GLUT5, and expressed at the brush border of the intestinal epithelium, as shown in Figure 1. GLUT5 is a facilitative transporter, which means that the transport of fructose into intestinal cells is proportional to the concentration gradient across the luminal membrane of intestinal cells (38). Although the expression of GLUT5 is responsible for intestinal fructose absorption, it is also expressed in other tissues and cell types, including skeletal muscle, pre-adipocytes, prostate, spermatozoa, and erythrocytes (39–42). However, the importance of fructose transport to these tissues is poorly understood. Exceeding the clearance capacity of the small intestine, excessive fructose escapes the small intestine and reaches the large intestine and liver (1).

### Fructose metabolism in intestinal epithelial cell

The small intestine is the major and first site for fructose metabolism, as shown in Figure 1 (1). Fructose concentration in peripheral plasma is typically about 0.04 mM, which is consistent with the data reported by the previous studies (27, 28, 43–45). By contrast, the concentration of fructose in the intestine (6 to 15 mM) is higher than peripheral plasma (39, 46). This observation provides evidence that dietary fructose metabolism started in small intestinal epithelial cells, and was formed by ketohexokinase (KHK) phosphorylation to form a fructose-1-phosphate (F1P), which accumulated in cells with high levels (1, 47). Much of the fructose is metabolized to lactate, alanine, glycerate, and other organic acids within the intestine. The small intestine fully express glucoisomerase to efficiently convert fructose into circulating glucose (1, 48, 49), which is the mechanism of maintaining liver health. Low doses of fructose(~0.5 g/kg) are 90% cleared by the intestine (1), but high doses of fructose (≥1 g/kg), exceeding intestinal fructose absorption and clearance, results in fructose reaching both the liver and colon, where it may cause disease by impacting hepatic function or microbial composition (1).

### Fructose metabolism in hepatocyte

It has been assumed that the liver is another vital site of dietary fructose metabolism (47, 50–52). In a more recent study, it was reported that the liver is able to extract 70% of the oral fructose load, whereas the liver extracts only 10% to 35% of the oral glucose load (53, 54). A small fraction of the carbon in ingested fructose is converted into glucose by the liver and enters the systemic circulation. These observations demonstrate that the rapid clearance of fructose in the blood is largely mediated by the effective extraction of the liver (21). When fructose reaches the liver from the portal vein, fructokinase phosphorylates fructose to fructose-1-phosphate, which can be converted to glycerol-3-phosphate for synthesis of glycerol or metabolized to acetyl-CoA and incorporated into fatty acids through *de novo* lipogenesis preferentially (55). In fact, fructose is converted to fatty acids at a higher rate in the liver than glucose (56). The entry of fructose into the lipogenic process may contribute to fructose-induced hyperlipidemia, particularly the marked increase in postprandial triglyceride levels (57, 58). Pathologically, recent studies have confirmed that NAFLD is commonly associated with excessive fructose intake (4, 59). Based on the cellular metabolism and function of fructose in the intestine and liver, investigations are required for in-depth study.

## Fructose consumption and metabolic roles in pigs

At present, most of the studies on fructose are from mouse model, which is widely used but not well-accorded with human diseases, so we have chosen to focus on another greater model than murine: pigs. Pig is a more suitable biomedical model for humans than mouse (60). The anatomical and physiological similarities between pigs and humans suggests pig model to be an important tool for medical research, providing important models for human therapies (61). In addition, pig production plays an important role in farming systems worldwide (62) and the large-scale pig industry is developing rapidly all over the world to satisfy the growing requirement of consumers (63). However, there are still some problems that are difficult to solve at present, for example, postnatal growth retardation (PGR) is common in piglets, which are prone to developing chronic disease (64). Therefore, we further summarized the roles of fructose in pig production and pig biomedical models for human metabolic diseases.

### Fructose in reproductive performance of pigs

Reproductive performance has a great influence on sow productivity and production profit (65). As mentioned in Table 1, McCracken (70) gave pre-pubertal gilts dietary treatments containing 35% fructose for 9 weeks, and found that the size of the reproductive tract had increased but the pregnancy rate decreased. It was also observed that fructose can increase litter size of primiparous sows but had no effect on individual weight and milk yield (67). However, there are different views that the milk yield of sows fed with a fructose diet increased significantly on day 14 and day 21 during a 22-d lactation, implying that fructose might be used to increase total yield of milk during lactation (66), but the dose and concentration need to be further studied. In fact, there is increasing clinical evidence that fructose contributes to elongated estrous cycles and hyperandrogenemia, accompanied by decreased LH concentrations and an increase in the number of follicles and in the level of luteal phase progesterone (68). Mechanically, Ossabaw pigs fed with fructose have increased transcript levels and dysfunctional steroidogenic enzymes in the ovarian delta 4 steroidogenic pathway, leading to hyperandrogenemia (69). However, the detailed mechanism of how fructose affects the reproduction functions of pigs needs further investigation.

**Table 1:** Role of fructose in pigs.

	**Subject**	**Fructose dose or markers**	**Duration**	**Outcome**	**Ref**
Reproduction	25 Hampshire x Yorkshire x Large White sows	24%	3 weeks	The milk yield of sows fed with a fructose diet increased significantly on the day 14 and day 21.	(66)
	45 crossbred sows and 36 gilts	20%	4 weeks	Fructose can increase litter size of primiparous sows but have no effect on individual weight and milk yield.	(67)
	9 multiparous Ossabaw female pigs	8.9%	8 months	Fructose contributes to elongating estrous cycles and hyperandrogenemia, accompanied by decreasing LH concentrations and increasing the number of follicles and the level of luteal phase progesterone.	(68)
	19 nulliparous Ossabaw miniature pigs	8.9%	8 months	Obese Ossabaw pigs have increased transcript levels and function of ovarian enzymes in the delta 4 steroidogenic pathway.	(69)
	Female crossbred pigs	35%	9 weeks	Fructose consumption increases reproductive tract size but reduces reproductive capabilities.	(70)
Development	16 crossbred fetal pigs	^14^C-fructose	–	Glucose acts as a precursor of fructose and converts to fructose in porcine endometrium and placenta.	(71)
	8 fetal pigs from two primiparous crossbred Yorkshire x Hampshire gilts	^14^C-fructose	–	Fructose is involved in the synthesis of nucleic acid and provides a substrate for the synthetic metabolic function needed for fetal growth and development.	(72)
	An established mononuclear porcine trophectoderm cell line from day 12 pig conceptuses	–	–	Fructose is mediated by the hexosamine biosynthesis pathway to stimulate mTOR cell signaling, proliferation of porcine trophectoderm cells, and synthesis of hyaluronic acid, a significant glycosaminoglycan in the pregnant uterus.	(73)
	8-month-old crossbred gilts	–	–	Conversion of glucose to fructose is present at the uterine-placental interface of pigs.	(74)
	8-month-old crossbred gilts	^14^C-fructose	–	Glucose and fructose transporters are precisely regulated in a spatial-temporal pattern along the uterine-placental interface of pigs to maximize hexose sugar transport to the pig conceptus/placenta.	(75)
Biomedical model	Juvenile female Ossabaw swine	4.45%	16 weeks	Pigs become obese, with adverse effects on liver, blood lipids, microflora, and a NAFLD phenotype.	(76)
	Female Göttingen Minipigs	20%	20 weeks	Fructose produces potential lipogenesis through precursors that can be used for DNL, leading to fat accumulation and liver steatosis.	(77)
	13-day old Iberian pigs	10 g fructose and 20.6 g fat	9 weeks	Fructose interferes with skeletal muscle metabolism and reduces fat metabolism in piglets.	(78)
	Male Danish Landrace × Yorkshire × Duroc pigs	60%	4 weeks	No macrovesicular steatosis or hepatocyte ballooning.	(79)
	Iberian pigs	64%	16 weeks	Increase of butyric acid synthesis, triglyceridemia, and subcutaneous fat deposition in young Iberian pigs. No differences in histological markers of NAFLD.	(80)

### Fructose metabolism in growth and development of pigs

Despite the controversial role of fructose in reproduction, fructose is the most abundant hexose sugar in pig embryos, indicating a specific function of fructose in embryo development. It has also been shown to be the principal blood sugar in the ungulate fetus (81–83). In fetal pig blood, the concentration of fructose is higher than glucose (84). Gluconeogenesis rarely occurs in the placenta and embryo of pigs (85). It means that glucose and fructose in maternal blood must be transported to the uterine cavity to ensure the use of glucose and fructose in the placenta and fetus. In pigs, glucose and fructose are transported from the maternal vascular system to the placental vascular system through multiple cell layers in a SLC2A-dependent manner (74, 75). Notably, intraperitoneal injection of sugars containing uniformly labeled carbon (UL14C) into pigs shows that glucose may act as a precursor of fructose and convert to fructose in porcine endometrium and placenta (71, 74). Due to the high concentration of fructose, a partial amount of fructose is involved in the synthesis of nucleic acids, thereby providing substrate for anabolic functions necessary for fetal growth and development (72). In a more recent study, it is reported that fructose can be used as an alternative energy source by embryos that express enzymes to promote fructose to enter the glycolysis pathway for metabolism (74). Moreover, it was observed that fructose is mediated *via* hexosamine biosynthesis pathway to stimulate mTOR cell signaling, which can promote embryonic/fetal growth and development (73). Thus, it is of importance to better understand the critical role of fructose in the placenta and embryo.

### Pig acts as a human biomedical model for fructose-induced metabolic disorders

Animal biomedical models play a central role in human medical research and developing new therapeutical strategies (61). Pig biomedical models for human reproduction have greatly advanced our understanding of the basic science of fertilization and pregnancy, as well as metabolic diseases including NAFLD (61). Although there are several factors that may lead to NAFLD, excessive consumption of fructose is considered to be a key factor in the formation of the disease (3–5). A recent study reported that juvenile Ossabaw swine fed a high-fat, high-fructose, high-cholesterol diet, containing 17.8% HFCS, can develop obesity and have serious effects on liver, blood lipids, microflora, and a NAFLD phenotype (76). Furthermore, a high-fructose and high-fat diet (HFF) interfered with the skeletal muscle metabolism of Iberian piglets after feeding for 10 weeks (78). Mechanically, there is increasing clinical evidence to show that fructose produces potential lipogenesis through precursors that can be used for *de novo* lipogenesis (DNL), leading to fat accumulation and liver steatosis (77, 86).

However, it has been reported recently that a high-fructose diet does not induce NAFLD. Some studies demonstrate that, compared with a sucrose-enriched diet, histological markers of NAFLD in Iberian pigs showed no significant increase in the fructose group (80). Feeding castrated male Danish Landrace-Yorkshire-Duroc pigs with 60% fructose for 4 weeks showed no steatosis or hepatocyte ballooning in the liver, providing evidence that short-term feeding with a high-fructose diet may not induce NAFLD in pigs (79). Collectively, breed and age of pigs might be factors that protect pigs from the development of steatosis.

## Conclusion and perspective

Over the last decade, studies have illustrated the role of fructose and its metabolic mechanism. However, the functional effect of fructose *in vivo* remains controversial, and there is no conclusion on the specific healthy use of fructose. Moreover, its different roles in pigs are challenging and worthy of exploration. In piglets, PGR is also prevalent in pig production (64). From the perspective of the effect of fructose, it indicates that fructose might have a potential growth-promoting effect. Besides, taking the safe thresholds for sugar consumption into account will be a matter of concern in nutrition and the detailed mechanism of how fructose affects the reproduction functions of pigs warrants further investigation. Hopefully, better understanding of the complex role of fructose may open new avenues for curbing human metabolic diseases and improve pig production.

## Author contributions

TS designed this review, helped with writing and revising of the manuscript, and provided critical feedback. JX and SS conceptualized the topic, researched and analyzed the literature, and composed and revised the manuscript. YL, SW, and SR helped draft the manuscript. All authors approve the final version of the manuscript, ensure the accuracy and integrity of the work, and agree to be accountable for all aspects of the work.

## Funding

This work was supported by grants from the National Natural Science Foundation of China (32102561), the Chinese Fundamental Research Funds for the Central Universities (2662019QD022), the Student Research Funds of Huazhong Agricultural University (S202110504033), National Innovation and Entrepreneurship Training Program for Undergraduate (202210504008), and the State Key Laboratory for Managing Biotic and Chemical Threats to the Quality and Safety of Agro-products (2021DG700024-KF202214).

## Conflict of interest

The authors declare that the research was conducted in the absence of any commercial or financial relationships that could be construed as a potential conflict of interest.

## Publisher's note

All claims expressed in this article are solely those of the authors and do not necessarily represent those of their affiliated organizations, or those of the publisher, the editors and the reviewers. Any product that may be evaluated in this article, or claim that may be made by its manufacturer, is not guaranteed or endorsed by the publisher.
